# Pharmaceutical Association

**Published:** 1878-12-20

**Authors:** 


### PHARMACEUTICAL ASSOCIATION.

The Pharmaceutical Association which assembled in this city on the 26th ult., was well attended by an intelligent body of Pharmaceutists. Our space does not admit of more than a glance at the interesting proceedings. Their cordial reception and the elegant repast given them through the efforts of the Pharmacists and Druggists of the city, seemed to be duly appreciated, eliciting warm and appropriate resolutions of thanks, &c. The discussions upon various queries pertaining to the preparation of drugs and the properties and qualities of indigenous plants, &c., were both interesting and instructive. In these discussions our friend Mr. J. U. Lloyd, of the firm of Merrell, Thorpe & Lloyd, of Cincinnati, disclosed much ready information and thorough knowledge upon all matters pertaining to Pharmacy, in which he, and the house with which he is connected, has acquired a deservedly high reputation. His exhibit of chemicals were beautiful, evincing neatness, care and special attention to the strength and purity of preparations.

We were struck also with the exhibit made by the following houses:

WM. R. WARNER & Co., in all respects—especially in the line of sugar-coated pills.

MCKESSON & ROBBINS, for the same, especially in the line of *Fluid Extracts* and gelatine coated pills.

Messrs. POWERS & WEIGHTMAN, a very large and elegant display.

PARK DAVIS & Co., the large and enterprising firm of Cincinnati, made also a very fine exhibit.

JAS. G. STEELE & Co., of San Francisco, Cal.; their exhibit attracted much attention, especially in the line of western indigenous plants.

H. R. MENSING'S Filtering Apparatus made a favorable impression.

HANCE BROS. & WHITE, Philadelphia, were on hand with a fine display.

JOHN WYETH & BRO., Philadelphia, were also represented, making a beautiful and varied exhibit of chemicals.

ROSENGARTEN & SONS, Manufacturing Chemists, Philadelphia.—An interesting advertisement from this excellent house commences in the present issue of our journal. Their display was truly excellent, particularly of the Cinchona alkaloids and the salts of morphia.

HENRY THAYER & Co., Cambridgeport, Mass., who also have an advertisement in present number of our journal, were represented. We were much pleased with their exhibit. Their Resinoids and Fluid Extracts, sugar coated pills, &c., are of a superior manufacture. They present a large list of new remedies. An old, well established and reliable house.

The Botanical display of our fellow citizen, Dr. Grant, speaks well for his information and energy in the department of Botany.

Upon the whole we were both interested and delighted with these exhibits as furnishing satisfactory evidence of a growing interest and rapid progression in the department of Pharmacy in our country.

